# Antiarrhythmic drug use in atrial fibrillation among different European countries – as determined by a physician survey^☆^

**DOI:** 10.1016/j.ijcha.2025.101709

**Published:** 2025-05-31

**Authors:** Espen Fengsrud, Carina Blomström-Lundqvist, A. John Camm, Andreas Goette, Peter R. Kowey, Jose L. Merino, Jonathan P. Piccini, Sanjeev Saksena, James A. Reiffel, Giuseppe Boriani

**Affiliations:** a Department of Cardiology, Örebro University Hospital, Faculty of Medicine and Health, Department of Medical Sciences, Örebro University, Örebro, Sweden; b Department of Medical Science & Cardiology, Uppsala University, Uppsala, Sweden; c Cardiovascular and Genetics Research Institute, St George’s, University of London, London, UK; d St Vincenz Hospital Paderborn, Department of Cardiology and Intensive Care Medicine, Paderborn, Germany; e Sidney Kimmel Medical College at Thomas Jefferson University, Philadelphia, and Lankenau Heart Institute PA, USA; f La Paz University Hospital, Idipaz, Autonoma University, Madrid, Spain; g Duke Clinical Research Institute, Duke University, Durham, NC, USA; h Rutgers Robert Wood Johnson Medical School, Piscataway, NJ, USA; i Columbia University, New York, NY, USA; j Cardiology Division, Department of Biomedical, Metabolic and Neural Sciences, University of Modena and Reggio Emilia, Policlinico di Modena, Modena, Italy

**Keywords:** Atrial fibrillation, Antiarrhythmic drug, Physician, Survey, Guidelines

## Abstract

**Background:**

There is limited knowledge of physicians’ antiarrhythmic drug (AAD) treatment practices for patients with atrial fibrillation and adherence to guidelines in European countries.

**Methods:**

An online survey (n = 321) of cardiologists, cardiac electrophysiologists and interventional electrophysiologists was conducted in Germany (DE; n = 83), Italy (IT; n = 95), Sweden (SE; n = 60) and the United Kingdom (UK; n = 83) including 96 questions on treatment practices.

**Results:**

ESC guidelines were the most important non-patient factor influencing treatment practice (55–72 %). However, while amiodarone was frequently (88–93 %) used in heart failure with reduced left ventricular ejection fraction, it was also a typical treatment choice for minimal/no-structural heart disease (SHD) (28 %), particularly in UK. Other deviations from guidelines were the use of class 1C drugs in coronary artery disease (CAD) and other SHD, and use of sotalol in left ventricular hypertrophy and renal impairment. In-hospital initiation of sotalol was low, with the exception of SE. Sotalol (16–41 %) and dronedarone use (10–54 %) in CAD varied among countries. For frequent, symptomatic paroxysmal AF, ablation was generally favoured, but AADs were preferred by 53 % in SE. In asymptomatic or subclinical AF, AADs were used by 41 % (range: 22–60 %), ablation by 11 % (range 2–18 %). In contrast to guidelines that prioritize safety, anticipated efficacy was more important (51 %) than safety (31 %) when selecting AADs.

**Conclusions:**

Despite recognizing the importance of guidelines, deviations in AAD use were common with the potential to compromise patient safety. These findings indicate the need for more educational support for optimal AAD selection in AF management.

## 1 Introduction

Atrial fibrillation (AF), the most common sustained cardiac arrhythmia, is associated with a five-fold risk of stroke [1], a three-fold risk of heart failure [2], a doubled risk of mortality [3], and individuals with AF are at increased risk of cognitive impairment and dementia [4]. Quality of life is often impaired, and hospitalizations are more frequent [5]. The prevalence of AF is increasing, predicted to be 18 million people in Europe by 2060. AF represents an increasing burden on the health care system.

The European Society of Cardiology (ESC) provides physicians with guidelines to direct the management of patients with AF [6]. Guidelines advocate the use of AADs and/or ablation for rhythm control in symptomatic AF and selection of antiarrhythmic therapies should consider severity of symptoms, arrhythmia burden, presence of underlying heart disease, and risk of adverse effects. During the last two decades, the management of AF towards a rhythm control strategy has evolved considerably in the guidelines. There have been advances in antiarrhythmic drug therapy, and novel one-shot catheter ablation tools for pulmonary vein isolation, have increasingly been used for rhythm control therapy [7]. However, prescribing practices of AAD and physicians' attitudes towards the management of patients with AF are still poorly understood. This study explored cardiologist and electrophysiologist treatment practices in patients with AF in four European countries (EU). Results are reported in the context of 2020 ESC guidelines.

## 2 Methods

### 2.1 Study design

This is a sub-study of the published AIM-AF survey, an exploratory, online physician survey, designed by a steering committee of nine global experts in AF [8]. Practicing physicians from the M3 Global International Market Research Panel were invited to complete the survey, with a geographical spread across four European countries, to avoid potential selection bias. Ethics approval was obtained from the local ethics committee in Uppsala, Sweden; participants provided informed consent in accordance with institutional guidelines.

### 2.2 Study population

The survey recruited 321 clinical cardiologists, including clinical electrophysiologists (EDs) and interventional electrophysiologists (EPs) from Germany (DE), Italy (IT), Sweden (SE), and the United Kingdom (UK). These countries were selected to ensure physicians from Central, Northern, Southern, and Western Europe were represented. Inclusion criteria were qualification in their specialty for > 3 years and < 40 years; >40 % of time actively treating patients; ≥30 new or existing patients with AF seen within a 3-month period and management of patients with AF who have received ablation or have been referred for ablation.

### 2.3 Data collection and analysis

The survey was conducted between October 2020 and February 2021 and was intended to take 60 min. Respondents were asked to complete 96 questions **(**Supplemental Table S1**),** including a set of screening questions to ascertain demographics and eligibility. Questions were grouped based on topics such as physician setting and patient caseload; treatment journey, with a focus on oral AADs; prescribing/treatment practices; and use of or referral for ablation. Survey questions were designed to understand physicians’ *general* approaches to the management of patients with AF and comprised closed questions, with a small number of open-ended questions to probe physician perceptions and behaviors.

The survey was performed in compliance with the European Pharmaceutical Market Research Association (EphMRA) code of conduct.

Univariate tests were conducted for comparisons between groups and the Z-test was applied to determine statistical significance (*P*-value boundary of < 0.05); however, no adjustment was made for multiple testing, so *P*-values may represent an overestimation of the statistical differences. It is important to emphasize that what we report in this survey is the proportion of respondents who may use a certain therapy in certain specific situations and not an actual prescribed therapy.

To distinguish the degree of non-adherence with recommendations from the 2020 ESC guidelines we established four definitions to describe adherence: compliance with guidelines (AAD use aligns with guideline recommendations); non-compliance with guidelines (AAD use contradicts guideline recommendations); deviation from guidelines (guidelines recommend use of an alternative therapy or alternative practice in this setting); and potential non-compliance with guidelines (use in this setting *could* contradict guideline recommendations, but clinical thresholds differed between the survey questions and the guidelines, preventing absolute certainty)**(**Fig. 2**).**

## 3 Results

### 3.1 Attitudes towards guideline adherence and physicians’ profiling

Guidelines were the first most important non-patient factor that influenced treatment strategy by 65 % of respondents (55–72 %). This was significantly more frequent in SE and IT (68 % and 72 % respectively) than in the UK (55 %), (p < 0.05) **(**Supplemental Fig. S1**).** Only 24 % reported that the 2020 ESC guideline influenced their survey responses while 58 % were unsure.

A total of 321 of 1980 (16 %) physicians approached completed the survey **(**Table 1**)**. The proportion of EPs were significantly higher in the other European countries (45 %) than in SE (20 %), (p < 0.05), and a specialization in AF was more frequent, 90 % vs. 58 % in SE, respectively, (p < 0.05). The most common clinical practice setting was a university hospital/clinic (49 %) but with some variation between countries **(**Table 1**).** Respondents had been qualified in their specialty for an average of 14 years. The average total cardiology patient caseload was 481 over a typical three-month period but varied significantly between countries (Table 1**).** Overall, most respondents prescribed AAD and referred patients for ablation rather than performing ablations themselves (58 %). However, while almost half of the respondents in IT, DE and UK (47 %) performed ablations this was the case for only 17 % in Sweden (p < 0.05) **(**Table 1**)**.

**Table 1:** Survey response, completion rates, physician profiling and demographics.

	Italy	Sweden	Germany	UK	Europe
**Invitations sent, n**	500	232	612	626	1980
Responses,*n (%)	177(35)	94(41)	121(21)	144(24)	543(27)
**Completed survey, n (%)**	95(19)	60(26)	83(13)	83(13)	32(16)

**Physician type, n (%)**					
*Cardiologist*	54(57)	48(80)	48(58)	42(51)	192(60)
*EP*	41(43)	12 (20)	35 (42)	41 (49)	129 (40)

**Sub-specialty, n (%)**					
*AF*	87(92)	35(58)	78(94)	70(84)	270(84)
*Other*	5(5)	14(23)	2(2)	9(11)	30(9)
*None*	3(3)	11(18)	3(4)	4(5)	21(7)

**Time qualified in specialty**					
*3–25 years, n (%)*	82(86)	56(93)	81(98)	81(98)	300(93)
*26–40 years, n (%)*	13(14)	4(7)	2(2)	2(2)	21(7)
*Mean, years*	14.9	15.0	13.4	12.9	14.0

**Time physician activities, %**					
*Treating patients*	87	84	87	84	86
*Academia/research*	9	7	6	8	8
*Administration/other*	4	9	7	8	7

**Main clinical practice setting, n (%)**					
*Community hospital/clinic*	55(58)	27(45)	30 (36)	16 (19)	128 (40)
*University hospital/clinic*	31(33)	29(48)	30(36)	66(80)	156(49)
*Primary outpatient practice*	0(0)	1(2)	18(22)	0(0)	19(6)
*Private hospital/clinic*	9 (9)	3 (5)	4 (5)	0 (0)	16(5)
*Other*	0(0)	0(0)	1(1)	1(1)	2(1)

**Patient caseload, 3 months, n**					
*Total cardiology caseload*	459	286	698	429	481
*New patients with AF*	96	51	112	85	105
*Follow-up patients with AF*	184	78	159	118	141

**Clinical activities, n (%)**					
*Prescribe AAD/perform ablation*	50(53)	10(17)	37(45)	37(45)	134(42)
*Prescribe AAD/refer ablation*	45(47)	50(83)	46(55)	46(55)	187(58)

EP indicates interventional electrophysiologist. AF, atrial fibrillation;

Due to rounding, not all percentages add up to 100.

* Respondents who started the survey, including those who did not complete all questions.

### 3.2 The choice of AAD in specific clinical settings

Across multiple comorbidity categories, 60 – 80 % selected amiodarone as a typical AAD choice in most settings despite other *first-line* recommendations in the guidelines apart from in heart failure. Sotalol use ranged from 16 % to 43 %, dronedarone from 10–54 %, while use of class Ic drugs was low in different comorbidity categories **(**Fig. 2**).**

**Fig. 1:**
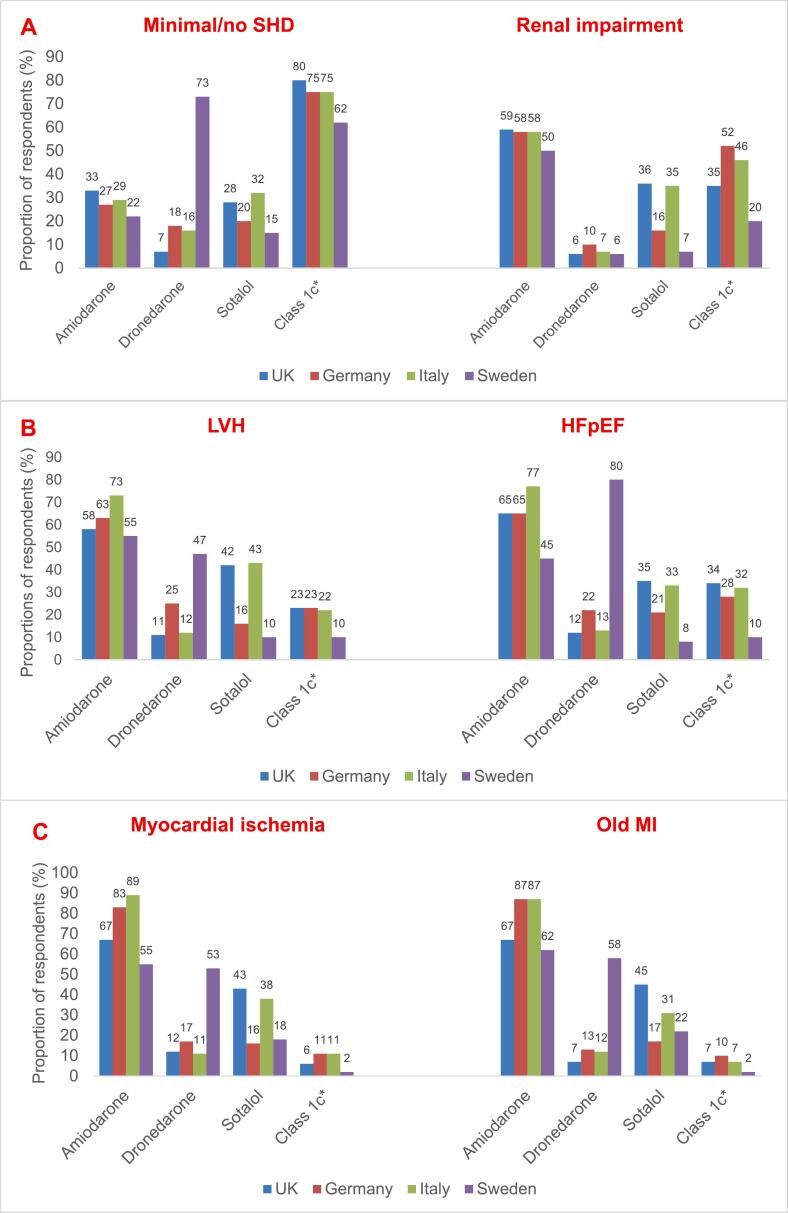

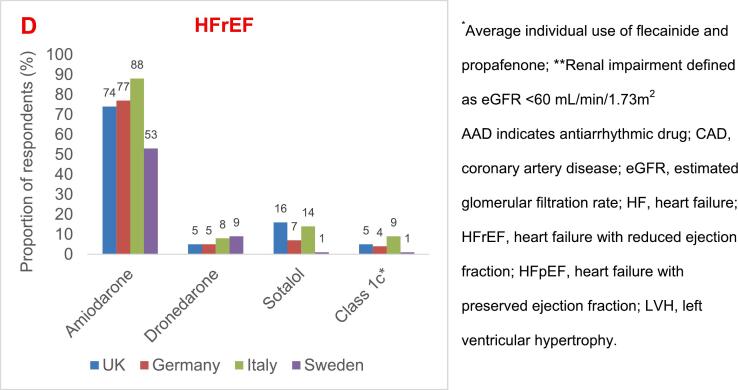
**Proportion of respondents who selected AADs as a typical treatment choice in patients with specific comorbidities.** (A) Patients with minimal/no SHD, renal impairment** (B) Patients with LVH and HFpEF. (C) Patients with CAD. (D) Patients with HFrEF. *Average individual use of flecainide and propafenone; **Renal impairment defined as eGFR < 60 mL/min/1.73 m2. AAD indicates antiarrhythmic drug; CAD, coronary artery disease; eGFR, estimated glomerular filtration rate; HF, heart failure; HFrEF, heart failure with reduced ejection fraction; HFpEF, heart failure with preserved ejection fraction; LVH, left ventricular hypertrophy.

In patients with *no or minimal structural heart disease* (SHD), 28 % selected amiodarone as a typical treatment option **(**Fig. 1**A)** in contrast to the guidelines, which recommend other AADs whenever possible. Sotalol use was overall 27 %; highest, 32 %, in IT and lowest, 15 %, in SE (p < 0.05) while dronedarone use was highest, 73 %, in SE vs. only 7–18 % in the other EU countries (p < 0.05).

**Fig. 2:**
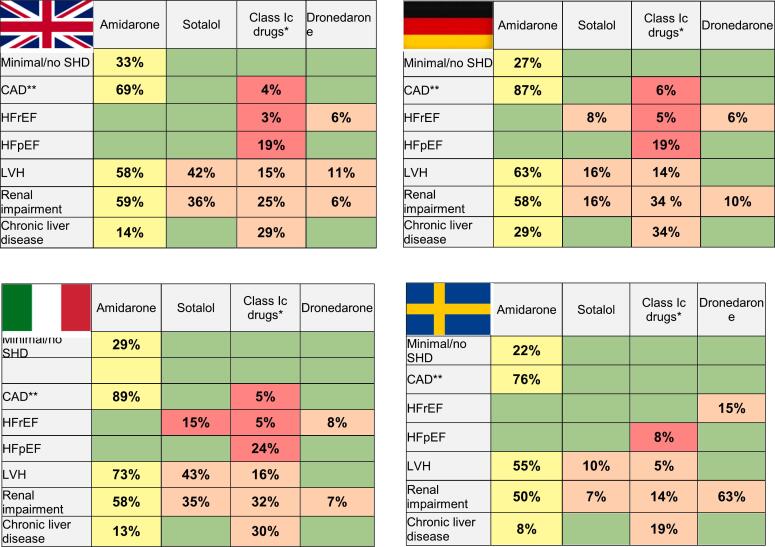
**Percentage of responders from all four countries who selected each AAD in specific clinical circumstances.** Data shown describe the percentage of respondents who selected each AAD in specific clinical circumstances. The color codes describe the degree of adherence / non-adherence between survey responses and the 2020 ESC guideline recommendations.^9^.*Average individual use of flecainide and propafenone;**Average use across myocardial ischemia, MI and revascularized CAD; AAD indicates antiarrhythmic drug; AF atrial fibrillation; CAD, coronary artery disease; HF, heart failure; HFpEF, heart failure with preserved ejection fraction; HFrEF, heart failure with reduced ejection fraction; LV, left ventricular; LVEF, left ventricular ejection fraction; LVH, left ventricular hypertrophy; MI, myocardial infarction; and SHD, structural heart disease. **Figure 2. will be complemented with name of country if needed.**

Although not guideline-recommended in patients with severe *left ventricular hypertrophy (LVH)*, sotalol and class Ic agents were typical treatment choices in these patients by 30 % (10 % in SE versus 43 % in IT, p < 0.05) and 13 % (5 % in SE versus 16 % in IT, p < 0.05), respectively **(**Fig. 1**B)**. In patients with *HF with preserved ejection fraction (HFpEF)*, guidelines do not recommend use of class Ic agents, yet they were selected by 18 % (24 % in IT vs 8 % in SE, p < 0.05) **(**Fig. 2**).**

The guideline recommended AAD for AF patients with CAD is primarily dronedarone (class IA) while sotalol has a class IIb LoE A recommendation. However, Class Ic agents were primarily selected in CAD by in average 6 % of respondents (Fig. 1**C)** (the exception being SE with only 2 % use **(**Fig. 2**)**).

Amiodarone is recommended for patients with *HF with reduced ejection fraction (HFrEF)* while dronedarone may be used in patients with mildly impaired, but stable LV function. Against guideline recommendations for HFrEF, sotalol was selected by 18 % (8 % in SE versus 24 % in IT, p < 0.05) and class Ic agents by 6 % (Fig. 1**D and 2)**.

In patients with *renal impairment* (eGFR < 60 mL/min/1.73 m^2^), sotalol was selected by 25 % which may be a potential non-compliance with ESC guidelines stating that sotalol should not be used if creatinine clearance (CrCl) is < 30 mL/min **(**Fig. 1**A).** Sotalol was more often used in IT (35 %) and UK (36 %) than in SE (7 %) and in DE (16 %), respectively, (p < 0.05)*.* **(**Table 2**).**

**Table 2:** Survey responses indicating significant* differences between proportions of different countries respondents reporting specific cases of guideline non-adherent practice.

Treatment practices n (%)	Germany (n = 83)	Italy (n = 95)	Sweden (n = 60)	UK (n = 83)	Europe (n = 321)
**HFrEF**					
*Class Ic*†	2(4)	5(5)	1(1)	5(3)	13(4)
*Sotalol*	7(8)	14(15)	1(2)	16 (19)	38(12)

**CAD**‡					
*Amiodarone*	72(87)	85(89)	35(58)	57(69)	248(77)
**LVH**					
*Class Ic*†	11(14)	15(16)	3(5)	12(17)	41(13)
*Flecainide*	19(23)	16(17)	6(10)	19(23)	60(19)
*Propafenone*	3(4)	14(15)	0(0)	5(6)	22(7)

**Chronic liver disease**					
*Amiodarone*	24(29)	12(13)	5(8)	12(14)	53(17)
*Dronedarone*	5(6)	5 (5)	8 (13)	3 (4)	21(7)
Class Ic †	28 (34)	33 (35)	12(19)	24 (29)	97(30)

**Renal impairment**‖					
*Class Ic*†	28(34)	32(34)	8(14)	20(24)	88(28)
*Sotalol*	13(16)	33(35)	4(7)	30(36)	80(25)
**Chronic lung disease**					
*Class Ic*†	39(47)	56(59)	19(32)	33(39)	146 (46)
*Sotalol*	13(16)	28(29)	5(8)	29(35)	75(23)

**Routine investigations**#**(at least annually)**					
**Hepatic function**					
*Dronedarone*	52(68)	60(65)	51(85)	43(56)	206(67)
**Renal function**					
*Sotalol*	44(57)	54(57)	26(49)	28(35)	152(50)

**Outpatient initiation of AAD**					
*Sotalol*	46(55)	72(76)	18(30)	76(92)	212(66)
*Flecainide*	47(57)	70(74)	12(20)	76(92)	205(64)

**Use of AADs for rhythm control**					
*Asymptomatic AF*	34 (41)	45 (47)	19 (32)	36 (43)	125 (39)
*Subclinical AF*	45 (54)	64 (67)	19 (32)	18 (22)	138 (43)

AAD,antiarrhythmic drug; AF, atrial fibrillation; CAD, coronary artery disease; LVH, left ventricular hypertrophy; HFrEF, heart failure with reduced ejection fraction.

† Average individual use of flecainide and propafenone.

# Total respondent numbers in in each country varied between drugs.

‖ Defined as eGFR < 60 ml/min/1.73 m^2^.

* P < 0.05.

‡ Average use across myocardial ischemia, MI and revascularized CAD.

### 3.3 Initiation and monitoring of AAD therapy

*Initiation* of sotalol outside a hospital setting was reported by 34 % with significant differences between countries, 92 % in UK and 30 % in SE (p < 0.05) **(**Table 2**) (**Supplemental Table S2**).** The corresponding figure for flecainide was 36 % (92 % in UK and 20 % in SE, respectively (p < 0.05)).

Guidelines recommend close monitoring of proarrhythmic risk factors in individuals using AADs. Respondents requested routine investigations (at least annually) most often for amiodarone **(**Supplemental Fig. S2**).** Electrocardiograms were routinely requested when using amiodarone, sotalol, and class Ic drugs by 87 %, 85 % and 84 %, respectively, of European respondents. Renal function was monitored by only 50 %. Overall, respiratory function monitoring was requested by 60 % when using amiodarone, while monitoring of hepatic function when using amiodarone, dronedarone, and class Ic was requested by 87 %, 67 %, and 29 % of all respondents, respectively.

### 3.4 Use of rhythm and rate control strategies across AF subtypes

**Fig. 3:**
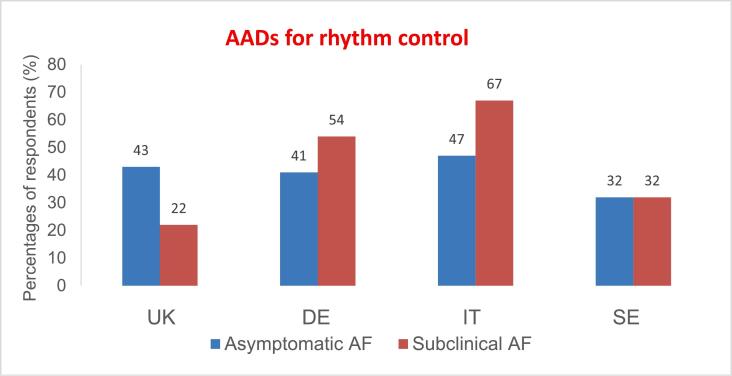
**AADs for rhythm control in asymptomatic and subclinical AF.** Proportion of respondents who described AADs in asymptomatic and subclinical AF. AAD indicates antiarrhythmic drug and AF atrial fibrillation.

**Fig. 4:**
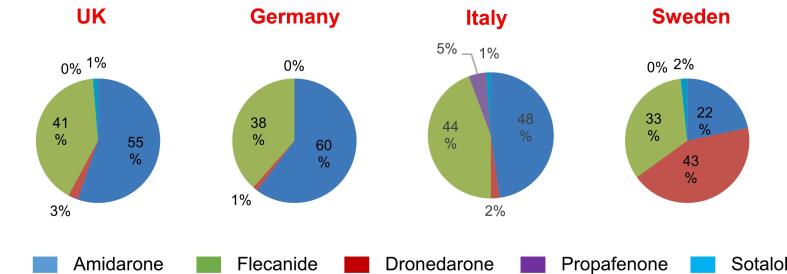
**Proportion of respondents who selected different AADs in combination with beta-blocker.** Proportion of respondents who selected different antiarrhythmic drugs in combination with beta-blocker.

Rhythm control as initial therapy was used by most respondents in patients with paroxysmal AF (PAF); although this was generally not the case for persistent AF (PeAF) (Supplemental Fig. S3**)**. Ablation was favored for patients with frequent, symptomatic PAF by most responders (65 %). However, AADs were preferred for both infrequent, highly symptomatic PAF (53 %) and frequent symptomatic PAF (53 %) in SE. Rhythm control therapies were also often selected for asymptomatic or subclinical AF **(**Fig. 3**)**. AADs were preferred by 41 % (22–60 %)of all respondents, while ablation was less frequently suggested 11 % (2–18 %), with both therapies more used in DE and IT vs UK and SE. Beta-blockers (94 %) were the most frequently suggested rate control agent in combination with an AAD for rhythm control, with some heterogeneity across different countries **(**Supplemental Fig. S4**).** Amiodarone was the AAD most frequently selected in combination with a rate control agent. Disparities between countries in preferred AAD in combination with beta-blocker are shown in Fig. 4. Dronedarone was the drug of choice significantly more often in SE than in the other countries (p < 0.05).

The ESC guidelines controversially recommend avoidance of combinations of AADs to minimize proarrhythmic risk. However, 22 % would try combinations of AADs (add-on) in patients with recurrence while receiving an AAD. In SE this was acceptable less often than in the other countries, 11 % vs 24 %, respectively (p < 0.05).

### 3.5 Factors influencing therapy selection

Despite guideline algorithms emphasizing safety first, efficacy was defined as the most important *non-patient factor* for selection of rhythm control therapy, while safety was considered the second most important factor Overall, 51 % (45–58 %) ranked efficacy first out of a list of nine general considerations with no statistical differences between countries **(**Supplemental, TableS3**).** Overall, 31 % ranked safety first (23 % in SE vs 42 % in DE, respectively, p < 0.05). The only group choosing safety first was EPs in SE (67 %). Symptomatic status was ranked by 38 % as the most important *patient factor* in guiding the choice of rhythm control therapy (data not shown). Regional differences regarding the degree of adherence to specific guideline recommendations were also observed **(**Table 2**).**

## 4 Discussion

The key finding from this study is that deviation from the 2020 ESC guidelines were notable in European practice. This was an unexpected result as 65 % of respondents stated that guidelines were the most important non-patient factor influencing their treatment decisions. The response rate seen was in line with those previously reported from online physician surveys [9,10]. The survey questions were phrased to ascertain general treatment practices, so respondents would not be expected to select answers based on individual patient circumstances. Some degree of non-adherence based on clinical judgment to adapt to individual patients should be expected and is of paramount importance in some situations. However, this survey-reported degree of deviation could indicate serious knowledge gaps among treating physicians, compromising patient safety [11]. In this context, it is notably that over half of the respondents considered anticipated efficacy (51 %) rather than safety (31 %) as the most important factor when selecting AAD therapy, contradicting current guidelines where safety has the highest priority. This could explain how respondents addressed some of the queries.

Despite the growing use of catheter ablation, appropriate use of AAD is an important issue in clinical practice [12,13,14]^.^ As the clinical presentations of AF evolve over time and guidelines are regularly updated in line with new evidence, patient safety can be compromised if physicians do not adapt to these updates. Further research to understand this behavior among clinicians and improvements in currently used methodology for guideline implementation are warranted. These observed disparities in safety concerns further indicates that a priority towards safety versus efficacy should be heightened in current guidelines. More educational support for optimal AAD treatment in AF should be emphasized.

Early published clinical trials such as Atrial Fibrillation Follow-up Investigation of Rhythm Management (AFFIRM) study and Rate Control vs, Cardioversion for Persistent Atrial Fibrillation (RACE) did not find significant differences in clinical outcomes when using rhythm control compared with rate control [15]. However, more recent data have shown greater improvement in quality of life, exercise tolerance, and reductions in both symptoms and symptomatic HF with restoration of sinus rhythm using AF ablation or AADs compared with rate-controlled AF [16,17,18]. The Early Treatment of Atrial Fibrillation Stroke Prevention Trail the (EAST-AFNET4) found that early rhythm control, including AAD and ablation, reduced the risk of adverse cardiovascular outcomes versus usual care, demonstrating that rhythm control remains a cornerstone for the treatment of many patients with AF [19], as was shown 10 years earlier in the ATHENA trial demonstrating survival benefits using dronedarone [20].

Despite this growing interest and evidence supporting early rhythm control, the ESC guidelines suggest limiting rhythm control to symptomatic patients only, unless there is a tachycardiomyopathy or heart failure. Our survey confirms, however, that rhythm control strategies are increasingly used for asymptomatic and subclinical AF across Europe with symptomatic status being the primary factor for choosing rhythm over rate control for only 38 % of participants. Certainly, the multiple recent studies suggesting a benefit of control of rhythm early in the course of AF may contribute to the use of rhythm control even in patients not yet significantly symptomatic. Similarly, since the years in which the CAST and other studies of that era demonstrated significant proarrhythmic risk of class Ic AADs in patients with CAD, some considerations may have changed. Early intervention in acute MI, interventional coronary artery procedures, use of cardiac calcium scans to define coronary artery lesions, which may not yet be of clinical significance, may all contribute to some physicians considering whether the restrictions on class Ic AADs should be less rigid.

Despite extracardiac toxicity and complex drug interactions, amiodarone was frequently chosen regardless of patient comorbidities. A high level of routine monitoring when using amiodarone, indicating awareness of its side effects. Our findings that class Ic drugs were used in a notable proportion of patients with CAD, HFrEF/HFpEF, or LVH, contradicts guidelines but does accord with the observations from the Outcomes Registry for Better Informed Treatment of Atrial Fibrillation (ORBIT-AF) [21]. In that study, 44 % of investigators used a class Ic agent in patients with CAD, which is against current guideline recommendations due to the potential risk of life-threatening proarrhythmia [22]. Moreover, 35 % used amiodarone as a first-line therapy in patients without HF or LVH. This was also observed in our survey.

In A sub analysis of the global observational REALISE-AF Survey description of amiodarone *first line*, class Ic or sotalol was not consistent with published guidelines in 50 %, 20 %, and 16 % of cases respectively. Adherence for AADs 2006 ESC guidelines was approximately 60 % [23].

In the EORP- AF registry adherence varied based on patient characteristics and comorbidities as observed in our study in [24].

When investigating the adherence to the ABC (Atrial Fibrillation Better Care) in the BALKAN-AF survey 44 % of patients were managed in adherence to the ABC approach. Interestedly, treatment by cardiologists was an independent predictor for adherent management [25,26].

These large registers are very important and helpful. However, data regarding prescriber treatment preferences are lacking.

Another important finding in our study was that Sweden deviated from the other countries, mainly in their more prevalent use of dronedarone. In the other countries sotalol was often selected in patients with LVH, renal impairment and HFrEF. This was also reported in the Get With The Guidelines study [27]. In the 2020 ESC guidelines [6], sotalol was downgraded from a class I to a class IIb LoE A recommendation based on the evidence of increased mortality compared with placebo and other AADs [28,29]. No specific recommendations with regards to In-hospital initiation of sotalol was included and initiation of AAD outpatient vs inpatient may be dependent of local regulations influencing the practice of hospitalizations. A substantial proportion of respondents were not influenced or were unsure if they were influenced by the 2020 ESC guidelines, which may explain the high use of sotalol across European countries studied, except for Sweden, where sotalol use was low and dronedarone use was high.

The high use of dronedarone in Sweden have several potential explanations. Sweden gradually changed from sotalol to betablockers for AF after A. Plewans [30] publication 2001 and the importance of ref. 22,28,29 in a small country favoured dronedarone before sotalol and flecainide. In addition, dronedarone was the recommended first line AAD by authorities after the expert committees' meetings, and many doctors adopted this. The discussion in Europe and now in the US after the publication of 2023 ACC/AHA/ACCP/HRS Guideline for the Diagnosis and Management of Atrial Fibrillation [31] with regards to sotalol, was never an issue in Sweden. The guideline adherence of AAD treatment was generally better in Sweden and more use of dronedarone could explain that amiodarone was less often preferred in CAD. However, respondents from Sweden were less likely to use ablation as first choice in AF treatment compered to respondents from the other countries. This may be explained by a long waiting list and relatively slow adaption to this well documented treatment.

The current study has extensively explored physicians’ attitudes towards antiarrhythmic therapies and their treatment practices in patients with AF, which provided a better understanding of physicians’ decision-making processes across Europe. Another strength of this study includes the fact that responses were collected from experienced cardiology physicians across several countries, the majority of whom considered AF to be their sub-specialty.

The new 2024 ESC guidelines [32] further emphasize rhythm control, particularly in patients who are symptomatic despite rate control. This includes a broader use of AADs general. Although limited data, also as a compliment to ablation therapy for optimal rhythm control [33,34]. Due to unchanged indications and contraindications for the individual AADs the importance of adherence to guidelines is crucial. In addition, after introduction of AF-CARE proper evidence based use of AADs should be spotlighted [35]. The recently published consensus document for AAD treatment may have an important impact [36].

## 5 Limitations

This survey report the proportion of respondents who *may* use a therapy in certain situations. However, there is practically no other way to get the opinion of the prescribing physican.and data regarding prescriber treatment preferences are lacking.

A key limitation is that data were dependent on the accurate reporting of information, which may have been subject to recall bias. The survey cohort was also limited to doctors within the M3 Global International Market Research Panel from only four European countries. The completion rate for the survey was 16 %, which while not atypical for such surveys, may not fully represent the broader physician population treating AF. However, it is likely that the responding physicians adhere to higher standards of care, which is noteworthy given the unsatisfactory guideline adherence observed. The study does not report gender-related data. Another limitation is the lack of AAD dosage considerations, which could affect both the safety and efficacy of treatment, potentially influencing the physicians’ responses.

Additionally, the set threshold values for specific queries diverged from the guidelines, making it difficult precisely to determine the number of non-compliant responders. The absence of dosing details and the inclusion of potentially non-adherent prescribing data may have contributed to a too high non-adherence rate for each medication, as either factor could lead to misclassifying adherent practices as non-adherent. Moreover, these rates do not account for the severity of deviation or the possible consequences of non-adherence.

## 6 Conclusion

Despite recognizing the importance of guidelines, and assertion that these are the primary determinant for rhythm control treatment, there was a high level of deviation from recommendations of the 2020 ESC guidelines of varying degrees with disparities among European countries. Further research to better understand drivers of non-adherence and more educational support for optimal AAD selection in AF is warranted in Europe. A priority towards safety versus efficacy should be emphasized in current guidelines.

## 7 Data access

Qualified researchers may request access to data including the study summary, study questionnaire with any amendments, and dataset specifications for validation purposes. Only fully anonymized data will be provided.

## Funding Sources

This study was funded by Sanofi. The funder had no role in either the study design, data collection, data analysis, data interpretation, or the decision to publish the study.

## CRediT authorship contribution statement

**Espen Fengsrud:** Writing – original draft, Visualization, Validation, Methodology, Investigation, Formal analysis, Conceptualization. **Carina Blomström-Lundqvist:** Writing – review & editing, Validation, Supervision, Methodology, Conceptualization. **A. John Camm:** Writing – review & editing, Visualization, Validation, Methodology, Formal analysis, Conceptualization. **Andreas Goette:** Writing – review & editing, Validation, Methodology, Conceptualization. **Peter R. Kowey:** Writing – review & editing, Visualization, Validation, Methodology, Conceptualization. **Jose L. Merino:** Writing – review & editing, Validation, Methodology, Conceptualization. **Jonathan P. Piccini:** Writing – review & editing, Validation, Methodology, Conceptualization. **Sanjeev Saksena:** Writing – review & editing, Validation, Methodology, Conceptualization. **James A. Reiffel:** Writing – review & editing, Validation, Methodology, Conceptualization. **Giuseppe Boriani:** Writing – review & editing, Validation, Supervision, Methodology, Conceptualization.

## Declaration of competing interest

The authors declare the following financial interests/personal relationships which may be considered as potential competing interests: [EF: No conflict of interest AJC: personal fees from Alta Thera, Sanofi, Abbott, Boston Scientific and Medtronic; CB-L: honoraria from Bayer, Boston Scientific, Correvio, Medtronic, Milestone, MSD (Merck & Co.),Sanofi and Pfizer; GB: speaker fees from Bayer, Boehringer Ingelheim, Boston and Medtronic; AG: speaker fees from Abbott, AstraZeneca, Berlin Chemie, Bayer, Bristol Myers Squibb-Pfizer, Boehringer Ingelheim, Daiichi-Sankyo, Medtronic, Novartis, Omeicos and Sanofi, and funding from the European Union Horizon 2020 (Grant No. 965286); PRK: consultant for Sanofi; JLM: personal fees from Boston Scientific, Microport, and Sanofi; JPP: receives grants for clinical research from Abbott, the American Heart Association, Boston Scientific, iRhythm, and Philips and serves as a consultant to ABVF, Abbott, AbbVie, Boston Scientific, ElectroPhysiology Frontiers, Kardium, Medtronic, Milestone Pharmaceuticals, Pacira, Sanofi, Philips, and Up-to-Date; SS: advisory board/research panel member for Sanofi and research grants from Abbott and Sanofi; JAR: investigator Janssen, Medtronic, and Sanofi and consultant Acesion, Amarin, Correvio, Medtronic and Sanofi.GB: speaker fees of small amount form Bayer, Boston Scientific, Abbott, Janssen, Sanofi].
