# Supplementary material for: Antiarrhythmic drug use in atrial fibrillation among different European countries – as determined by a physician survey
Source: Int J Cardiol Heart Vasc. 2025 May 31;59:101709. doi: 10.1016/j.ijcha.2025.101709 (PMC12164222; doi:10.1016/j.ijcha.2025.101709)
Supplement: Supplementary Data 2 [file mmc2.docx]

**Supplementary Material**

**Figures:**

**Figure S1. Most important non-patient factor that influenced treatment strategy**

Figure S1: Respondents first most important non-patient factor when choosing rhythm control before rate control

**Figure S2. Routine investigation**

**Figure S3.** Proportion of respondents who routinely (at least annually) request investigations in patients with AF receiving AADs. Illustrated by drug and country.ECG; electrocardiography, AAD; antiarrhythmic drug.

**Figure S3. AAD in different subtypes of AF**

AF; atrial fibrillation, AAD; antiarhrytmic drugs

**Figure S4. Preferred AADs and rate control drug combinations in different countries**

AADs; antiarrhythmic drugs

**Tables:**

| Outpatient initiation of AAD n (%) |  | UK  (n=83) | Germany (n=83) | Italy (n=95) | Sweden (n=60) |
| --- | --- | --- | --- | --- | --- |
| *Amiodarone* |  | 53 (64) | 33 (40) | 44 (46) | 39 (65) |
| *Dronedarone* |  | 56 (67) | 55 (66) | 47 (49) | 53 (88) |
| *Flecainide* |  | 76 (92) | 47 (57) | 70 (74) | 12 (20) |
| *Propafenon* |  | 62 (75) | 48 (58) | 64 (67) | 13 (23) |
| *Sotalol* |  | 76 (92) | 46 (55) | 72 (76) | 18 (30) |

**Table S1: Questionnaire (separate file)**

**Table S2: Outpatient initiation of AADs in different countries**

AADs; antiarrhythmic drugs.

**Table S3 First rank non-patient factor for selection of rhythm control therapy**

| *First ranked non-patient factor when choosing a particular AAD* | UK | Germany | Italy | Sweden |
| --- | --- | --- | --- | --- |
| *Efficacy* | 51% | 58% | 48% | 45% |
| *Safety* | 29% | 23% | 34% | 42% |
| *No hospital initiation* | 2% | 2% | 2% | 5% |
| *Comfort/ expirience with the drug* | 7% | 5% | 7% | 3% |
| *Need for ongoing ECG/lab. monitoring* | 4% | 4% | 1% | 0% |
| *Patient comorbidities* | 2% | 6% | 5% | 5% |

AAD; antiarrhythmic drug.
